# Inhibition of Melanoma Angiogenesis by Telomere Homolog Oligonucleotides

**DOI:** 10.1155/2010/928628

**Published:** 2010-06-28

**Authors:** Christina Coleman, Danielle Levine, Raj Kishore, Gangjian Qin, Tina Thorne, Erin Lambers, Sharath P. Sasi, Mina Yaar, Barbara A. Gilchrest, David A. Goukassian

**Affiliations:** ^1^Department of Dermatology, Boston University School of Medicine, Boston, MA 02118, USA; ^2^Feinberg Cardiovascular Research Institute, Feinberg School of Medicine, Chicago, IL 60611, USA; ^3^Division of Cardiovascular Research, Department of Medicine, Center of Cancer Systems Biology, Caritas St. Elizabeth's Medical Center of Boston, MA 02135, USA

## Abstract

Telomere homolog oligonucleotides (T-oligos) activate an innate telomere-based program that leads to multiple anticancer effects. T-oligos act at telomeres to initiate signaling through the Werner protein and ATM kinase. We wanted to determine if T-oligos have antiangiogenic effects. We found that T-oligo-treated human melanoma (MM-AN) cells had decreased expression of vascular endothelial growth factor (VEGF), VEGF receptor 2, angiopoeitin-1 and -2 and decreased VEGF secretion. T-oligos activated the transcription factor E2F1 and inhibited the activity of the angiogenic transcription factor, HIF-1*α*. T-oligos inhibited EC tubulogenesis and total tumor microvascular density matrix invasion by MM-AN cells and ECs in vitro. In melanoma SCID xenografts, two systemic T-oligo injections decreased by 60% (*P* < .004) total tumor microvascular density and the functional vessels density by 80% (*P* < .002). These findings suggest that restriction of tumor angiogenesis is among the host's innate telomere-based anticancer responses and provide further evidence that T-oligos may offer a powerful new approach for melanoma treatment.

## 1. Introduction

Angiogenesis, the formation of new blood vessels, is essential for tumor growth and metastasis [1] and inhibition of angiogenesis is an important new approach for therapy of many cancers [1, 2]. A principal regulator of angiogenesis is vascular endothelial growth factor (VEGF) [3]. The VEGF family is comprised of at least five genes (VEGF A through E), of which the most potent activators of angiogenesis are VEGF-A and VEGF-B [4]. Analysis of the VEGF-A (termed VEGF here and elsewhere in the text) gene promoter region has revealed numerous potential transcriptional activator sites [4]. One of the best studied stimuli for VEGF synthesis and secretion is hypoxia, which acts by upregulating hypoxia inducible factor-1*α* (HIF-1*α*) [5]. Inhibition of HIF-1*α* may therefore decrease angiogenesis by reducing VEGF levels and potentially other proangiogenic factors, such as angiopoeitin-1 and -2 (Ang-1 and Ang-2) [6–8], basic fibroblast growth factor (bFGF), and platelet derived growth factor (PDGF), all well known regulators of angiogenesis [1].

The role of E2F1 in apoptosis is well recognized. It can act in concert with p53 or independent of p53 to induce apoptosis [9]. T-oligos, telomere homolog oligonucleotides, activate p53 and E2F1, resulting in apoptosis [10]. However, in p53 null cells like malignant melanoma AN cells (MM-AN), apoptosis is induced by the p53 homolog p73 [11], presumably acting coordinately with E2F1. Moreover, E2F1 has also been reported to inhibit angiogenesis [9, 12]. While E2F1 decreases VEGF production in fibroblasts through p53 activation [12], its effect on angiogenesis in cells that lack functional p53, such as MM-AN cells has not been studied [11, 13].

Several lines of evidence suggest that normal cells have an integrated program of genome-protective responses, functionally analogous to the bacterial SOS response [14], that is based in the telomeres and appears to be abrogated in malignancy [15]. Telomeres, the ends of chromosomes, are maintained in a loop configuration by insertion of the single-stranded 3′ overhang into the proximal telomere duplex [16]. Disruption of this loop structure by removal of the principal binding protein TRF2 (telomere repeat binding factor 2) leads to exposure and digestion of the overhang and activation of ATM (ataxia telangiectasia mutated) and its effector protein p53, followed by apoptosis or senescence, depending on cell type [16]. Knockdown of another telomere-associated protein, the protection of telomeres-1 (POT-1), also expected to expose the TTAGGG telomere repeat sequence, activates ATR (ataxia telangiectasia and Rad3-related), leading to similar downstream effects [16, 17]. Moreover, treatment of malignant cells with RNAi to knockdown the expression of TER, the RNA subunit of telomerase, rapidly alters the expression of many genes in a pattern predicted to reduce cancer cell proliferation and invasiveness [18], then leads to apoptosis in a time course far too rapid to be attributable to the expected loss of telomerase activity and consequent telomere shortening, suggesting that other telomere-based effects are responsible [18].

Our laboratory has described several anticancer properties of oligonucleotides homologous to the telomere repeat sequence TTAGGG (T-oligos) [10, 11, 13, 15, 19–28]. T-oligos provided to cultured cells rapidly accumulate in the nucleus and mediate DNA damage responses without digestion of the telomere overhang or other detectable effects on genomic DNA [10, 13, 15, 19, 23, 25, 26, 29]. T-oligos activate the ATM kinase [21, 27], upregulate and activate p53 [30, 31], as well as upregulate and/or activate its homolog p73, E2F1, p16^INK4a^, p33, p27, and p95/Nbs1, and phosphorylate the histone variant protein H2AX [11, 13, 21, 22, 24, 25]. In addition, T-oligos promote differentiation of melanoma cells and downregulate the inhibitor of apoptosis protein IAP/livin in these cells [13]. T-oligo effects require WRN [22], the protein mutated in the progeroid cancer-prone Werner syndrome [32], and are associated with formation of classic DNA damage foci at telomeres [22]. In combination, these signaling cascades result in induction of apoptosis, autophagy, and/or senescence selectively in cancer cells [13, 19, 27, 28]; while in normal cells they lead to transient cell cycle arrest, increased DNA repair capacity and adaptive differentiation [19, 21, 23, 25–27, 30]. Complementary, unrelated, or scrambled oligonucleotides comparably accumulate in the nucleus, but do not cause DNA damage-like signaling or affect growth, differentiation or survival of malignant cells [11, 13, 15, 19, 21, 24, 27, 28].

Because the T-oligo-induced transcription factors p53, p73, and E2F1 are known to affect endothelial cell (EC) survival, differentiation, and proliferation during tumor angiogenesis [12, 33, 34] and because blocking angiogenesis would be an additional anticancer mechanism of action for T-oligos, we asked whether T-oligo treatment inhibits tumor angiogenesis. We now report that T-oligo inhibits angiogenesis in the aggressive human melanoma cell line MM-AN, derived from a metastatic melanoma [35], by decreasing production and secretion of proangiogenic factors in both tumor cells and ECs. As well, T-oligo treatment decreases the number of total and functional (perfused) vessels in flank tumors of MM-AN cells in SCID mice after two systemic injections.

## 2. Materials and Methods

### 2.1. Cell Cultures

Human microvascular endothelial cells (HMVECs) and human umbilical vein endothelial cells (HUVECs) were obtained at passage 2 and used by passage 4–6. Cells were maintained in EGM-2 medium with 2% FBS plus growth factors (bullet kit) (Cambrex Bio Sciences, Walkersville, MD). Human melanoma MM-AN and EP cells [35] were cultured in modified Eagle's medium (MEM) (Mediatech, Inc., Herndon, VA) supplemented with fetal bovine serum (FBS 2%), calf serum (CS 8%), and antibiotic-antimicotic. Human breast adenocarcinoma (MCF-7) and ovarian adenocarcinoma (OVCAR3) cells were cultured and maintained according to ATCC recommendations. All cells were incubated at 37° with 5% CO_2_.

### 2.2. Oligonucleotides

We used a 16-base 100% telomere homolog with the sequence 5′-GTTAGGGTTAGGGTTA-3′ and phosphodiester backbone. The oligonucleotide was synthesized by Midland Certified Reagent (Midland, TX) and then diluted from a 2 mM stock in medium to obtain a final concentration of 40 *μ*M.

### 2.3. Quantitative Real-Time Polymerase Chain Reaction (qRT-PCR)

MM-AN, EP, MCF-7, and OVCAR3 cells were grown in their corresponding media and treated once with diluent or 40 *μ*M T-oligo. For qRT-PCR, we performed a reverse transcription reaction then amplified specific genes as described [36]. mRNA expression levels were assessed for E2F1, ANG-1, and ANG-2. The sequences used were as follows: E2F1 forward: CGGTGTCGTCGACCTGAACT, E2F1 reverse: AGGACGTTGGTGATGTCATAGATG, E2F1 probe: TGCCGAGGTGCTGAAGGTGCAG; ANG-1 forward: CAGAAAACAGTGGGAGAAGATATAACC, ANG-1 reverse: TGCCATCGTGTTCTGGAAGA, ANG-1 probe: CAACATGGGCAATGTGCCTACACTTTC; ANG-2 forward: GGCTGGGCAATGAGTTTGTC, ANG-2 reverse: CCCAGTCCTTCAGCTGGATCT; ANG-2 probe: ACCGGTCAGCACCGCTACGTGC.

### 2.4. Electromobility Shift Assay (EMSA)

Cells were treated with diluent or 40 *μ*M T-oligo and harvested 0, 1, 16, and 32 hours after treatment. EMSAs were carried out as described [10] using a total of 5 *μ*g of nuclear protein per lane. The assay was performed using consensus sequences of E2F1 and HIF-1*α* transcription factors (Santa Cruz Biotechnology, CA). The specificity of the bands was confirmed by using either ×25 or ×50 excess of cold probe as competitor and mutant oligos as control. As an additional negative control, nuclear extracts were incubated with a specific competing E2F1 antibody before adding the radioactive (^32^P-labelled) consensus oligonucleotides.

### 2.5. Western Blot Analysis

Cells were treated with T-oligos, harvested at various times, snap-frozen, and stored at −70°C. Total cellular protein was isolated and 50 *μ*g of total protein was processed for western blot analysis as described [10]. Antibody reactions were performed with the following antibodies: anti-E2F1 (Neomarkers, Inc., Fremont, CA), anti-VEGF-A (sc-507, Santa Cruz Biotechnology, Inc, Santa Cruz, CA) that recognizes all VEGF-A isoforms, and anti-VEGF-R2 (a kind gift from Dr. Nader Rahimi, Departments of Ophthalmology and Biochemistry, Boston University School of Medicine).

### 2.6. VEGF Enzyme-Linked Immunosorbent Assay (ELISA)

The Human VEGF Immunoassay kit (Quantikine, R&D Systems, Minneapolis, MN) was used to compare the release of VEGF into the medium by ECs and MM-AN cells. Each cell type was cultured in its appropriate medium and treated with either diluent or T-oligo (40 *μ*M). The conditioned medium was collected at 24, 48, and 72 hours after treatment and frozen at −70°C until it processed for ELISA. The assay was performed according to the manufacturer's protocol (R&D Systems). The plate was read using the Tecan Spectra model 96 Well Microplate Reader (MTX Lab Systems, Vienna, VA).

### 2.7. Tubulogenesis Assay

200 *μ*L of Matrigel basement membrane matrix (phenol-red free) (BD Biosciences, Bedford, MA) was added per 4-well chamber slide and allowed to solidify for 1 hour at 37°C. HUVECs (50,000 cells) were then added to each chamber in 500 *μ*L of medium as described [37]. Cells were treated at the time of plating with diluent, 40 *μ*M, 80 *μ*M, or 120 *μ*M of T-oligo. Some of the cells treated with 40 *μ*M of T-oligo were retreated a second time at 2 hours postplating, at the time tube-formation could first be detected by phase microscopy. Some cells that were treated twice with 40 *μ*M of T-oligo received a third treatment 6 hours after plating. Tube-formation was observed and photographed at numerous time points starting from 2 hours and up to 24 hours after treatment. Of note, after tubular structures formed in control wells, they stayed intact up to 72 hours (data not shown). Image-Tool (The University of Texas Health Science Center in San Antonio, San Antonio, TX) software was used to quantify the length of tubular structures.

### 2.8. Invasion Assay

The BioCoat Matrigel Invasion Chamber (BD Biosciences, Bedford, MA) was used for this assay. MM-AN cells were treated with diluent or 40 *μ*M T-oligo and media were collected after 72 hours, based on ELISA data (MM-AN conditioned media). Conditioned MM-AN medium was then used as the chemoattractant for the assay. ECs and MM-AN cells were grown on the inserts (upper chambers) and allowed to invade through the Matrigel and attach to the membrane as described in the manufacturer's protocol. Invasion was assessed after 22 hours by staining with Diff-Quick (Fisher, Atlanta, GA) bisected membranes from the bottom of the chambers (containing the invading cells) as described in the manufacturer's protocol. Of note, we believe that any effect on ECs or MM-AN invasion cannot be attributed to residual active T-oligos in the conditioned medium, diffusing into the upper chamber and killing MM-AN cells, because T-oligos are rapidly degraded in serum-containing medium. With a measured half-life for a 12-base telomere homolog of 4–6 hours [38], the 16-base T-oligo would thus have been present in the medium after 72 hours of conditioning at an estimated (1/2)^12^ or 10^7^-fold less than the initial therapeutic concentration.

### 2.9. SCID Melanoma Flank Xenograft Model

All studies were approved by Boston University's IACUC Committee. 2 × 10^6^ MM-AN cells were injected subcutaneously into the flank of 6 week old SCID mice (Fox-Chase Cancer Center, Philadelphia, PA). Mice (5-6 per treatment group) were injected via tail vein with T-oligo or diluent alone when tumors were first palpable (2-3 mm diameter). Tumor sizes were recorded every 2-3 days throughout the experiment using electronic callipers and all animals were sacrificed 4 weeks after tumor inoculation. The diagnosis of melanoma was confirmed histologically on sections of each nodule cut through the center of the clinical tumor.

### 2.10. Quantification of Tumor Microvessel Density

All studies were approved by Boston University's IACUC Committee. 2 × 10^6^ MM-AN cells were injected subcutaneously into the flank of 6 week old SCID mice (Fox-Chase Cancer Center, Philadelphia, PA). Then mice (5-6 per treatment group) were injected via the tail vein with T-oligo or diluent alone (60 nmoles/injection, 15 mg/kg) when tumors were first palpable (2-3 mm diameter). Microvascular density (MVD) in bisected tumors were assessed 24 hours after two systemic injections of T-oligos by using two EC-specific markers CD31 (PECAM-1) [12] and *Bandeurea simplicifolia* (BS)-1 lectin conjugated to Rhodamine (Vector Laboratories, Burlingame, CA) [39]. To measure functional MVD in the tumor tissue, 30 minutes before sacrifice a set of mice (5 per treatment group) were anesthetized and were perfused with 0.5 mg (in 100 *μ*l of isotonic solution) of Rhodamine-conjugated BS-1 lectin as described [40, 41]. To measure total MVD, 6 *μ*m cross-sections of bisected tumor tissue (of the same mice perfused with BS-1 lectin) were stained with CD31 primary antibody followed by FITC-labelled secondary antibody as described [12]. Samples were photographed using a multicolour fluorescence microscope (Nikon, Nikon Instruments Inc, Melville, NY), and analyzed using a digital image analysis system (Nikon). The diagnosis of melanoma was confirmed histologically in tumor sections of each nodule.

### 2.11. Statistical Analysis

ANOVA with post hoc analysis by Scheffe and Bonferroni-Dunn and unpaired *t*-Test were performed using StatView (SAS Institute, Inc., Version 5.0). Statistical significance was established at *P* < .05. 

## 3. Results

### 3.1. T-oligo Treatment Decreases the Expression and Level of Angiogenic Factors and Reduces VEGF Release into MM-AN Cell Medium

**Figure 1:**
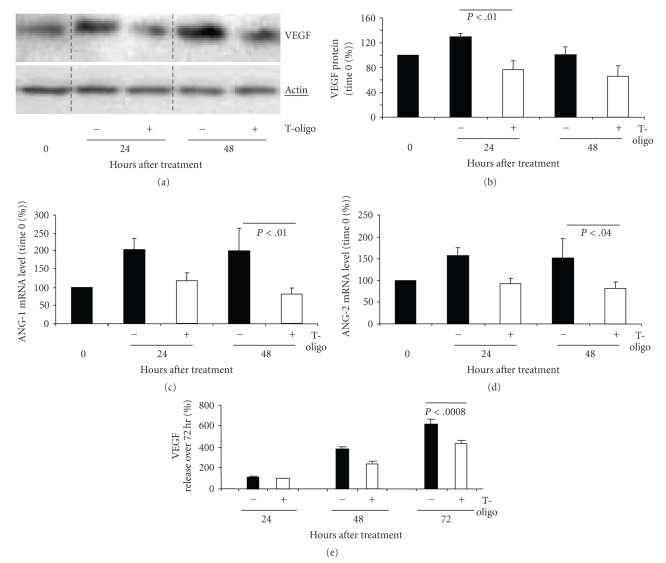
T-oligo treatment down-regulates expression of VEGF, Ang-1, Ang-2 and decreases VEGF production in MM-AN cells. MM-AN cells were treated once at time 0 with either 40 *μ*M T-oligo or diluent alone. (a) Western blot analysis of VEGF protein level. (b) Densitometric analysis of VEGF protein levels (after loading adjustment against actin expression) represented as a percent of time 0 level. Graphs represent pooled data (mean ± SEM) from three independent experiments. (c) and (d) Quantitative real time-PCR (qRT-PCR) of MM-AN cells treated with either 40 *μ*M T-oligo or diluent alone. Results are presented as percent of time 0 (set at 100%) and examined over 48 hours for both control and T-oligo-treated cells. ANG-1 gene expression. (c) ANG-2 gene expression. (d) These experiments are repeated twice with similar results. (e) MM-AN cells were treated with 40 *μ*M T-oligo or diluent alone. The culture medium was collected after 24, 48, and 72 hours. Cumulative VEGF protein released into the medium was measured by ELISA. Results represent data pooled from triplicate dishes for each time point and treatment condition. Changes over time are calculated as percent of the 24 hours values (set at 100%).

MM-AN cells were treated once with T-oligo or diluent alone, provided in fresh medium, and processed for western blot analysis. Compared to diluent, T-oligo decreased VEGF protein level at 24 hours (*P* < .01) by ~41% and at 48 hours (*P* = NS) by ~32% (Figures 1(a) and 1(b)). In addition, after 48 hours T-oligo treatment reduced ANG-1 mRNA (*P* < .01) by ~60% (Figure 1(c)) and ANG-2 mRNA (*P* < .04) by ~45% (Figure 1(d)). Similar decreases in VEGF (Supplemental Figures 1(a)–1(c) and ANG-1 (Supplemental Figures 2(a)–2(c)) mRNA were seen in a second human melanoma line EP, as well as in other malignant cell types.

Paired dishes of MM-AN and ECs were treated with 40 *μ*M T-oligo or diluent alone once. Medium was collected after 24, 48 and 72 hours and VEGF level in the medium was measured by ELISA. In ECs (HMVEC and HUVEC), there was a ~50%–75% variable and statistically insignificant decrease in VEGF release into the medium over time in both diluent- and T-oligo-treated cells (data not shown). In contrast, total VEGF release from MM-AN cells in diluent-treated dishes rose progressively over 72 hours to ~600% (of 24-hours VEGF levels), while VEGF release in T-oligo-treated dishes was decreased (*P* < .0008) by ~30% over the 72-hours experiment after a single dosing at time 0 (Figure 1(e)). Thus, T-oligo not only decreased VEGF protein expression in tumor cells but also the release of this potent angiogenic factor into the surrounding culture medium.

### 3.2. T-oligo Upregulates E2F1 Gene Expression, Protein Level, and DNA Binding Activity and Decreases the DNA Binding Activity of the Angiogenic Transcription Factor HIF-1*α*

**Figure 2:**
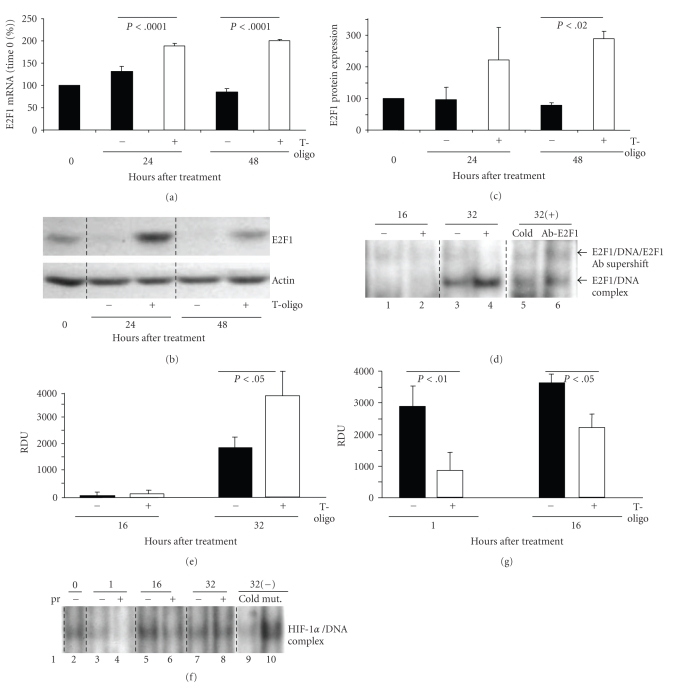
T-oligo treatment increases E2F1 expression/activity and decreases HIF-1*α* DNA binding activity in MM-AN cells. Cells were treated with 40 *μ*M T-oligo or diluent alone and harvested at various times. (a) The pellets were examined by qRT-PCR for E2F1 mRNA level, shown as a percent of time 0 levels (mean ± SEM) for 2 separate experiments in triplicate. (b) The pellets were also examined by western blot analysis for E2F1 protein levels. Actin expression was used as an internal loading control. (c) Densitometric analysis of E2F1 protein expression after loading adjustment by actin, represented as a percent of time 0 levels. Results are pooled data (mean ± SEM) from three independent experiments. (d) The DNA binding activity of E2F1 was analyzed by EMSA. No difference in E2F1 DNA binding activity was detected between the treatment groups at 16 hours (lane 1 versus 2) but E2F1 DNA binding activity doubled in T-oligo treated cells at 32 hours (lane 3 versus 4). Specificity of bands was confirmed by preincubating the nuclear protein of T-oligo-treated cells harvested at 32 hours with ×25 cold probe (lane 4 versus 5) and by supershift of E2F1 protein/DNA and E2F1 competing antibody complex (lane 4 versus 6). (e) Quantification of the band intensity of DNA binding activity is represented as relative density untis (RDU) for both treatment groups at 16 and 32 hours after treatment. E2F1 EMSA was repeated 2 times with similar results. **(**f**) **Nuclear protein was isolated from cells and processed for electromobility shift assay (EMSA) for evaluation of HIF-1*α* DNA binding activity. Specificity of the bands was confirmed by preincubating the nuclear protein extract of cells treated with diluent for 32 hours with ×25 cold probe (not labelled with ^32^P) and mutant HIF-1*α* consensus sequence for 20 minutes before incubating the nuclear extracts with ^32^P-labeled consensus oligonucleotides. HIF-1*α* EMSA was repeated 2 times with similar results. (g) Densitometric analysis of the protein/DNA complex bands for HIF-1*α* is graphed as relative densitometric units (RDU).

E2F1 mRNA expression was increased at 24 hours (*P* < .0001) by ~41% and at 48 hours (*P* < .0001) by ~100% in T-oligo-treated MM-AN cells compared to control (Figure 2(a)). Similar increases in E2F1 mRNA were seen in a second human melanoma line EP, as well as in other malignant cell types **(**Supplemental Figures 3(a)–3(c)). T-oligo treatment also increased E2F1 protein level at 24 hours to ~218% and at 48 hours to ~286% (*P* < .02) of control values (Figures 2(b) and 2(c)). In addition, there was a doubling in E2F1 DNA binding activity 32 hours after treatment compared to diluent-treated cells (Figures 2(d) and 2(e), compare lane 3 versus 4).

MM-AN cells were treated once with T-oligo or diluent alone and nuclear proteins were harvested up to 32 hours after treatment to evaluate DNA binding activities of the angiogenic transcription factor HIF-1*α*. There was ~80%–95% decrease in HIF-1*α* DNA binding activity at 1 and 16 hours after treatments (Figures 2(f) and 2(g), lanes 3 versus 4, and 5 versus 6, respectively). These data suggest that T-oligo-mediated inhibition in DNA binding activity of HIF-1*α* transcription factor contributes to decreased expression of the angiogenic factors, VEGF and ANG-1, whose promoters contain binding sites for HIF-1*α* [42].

### 3.3. T-oligo Inhibits Matrigel Invasion by Melanoma Cells and ECs

**Figure 3:**
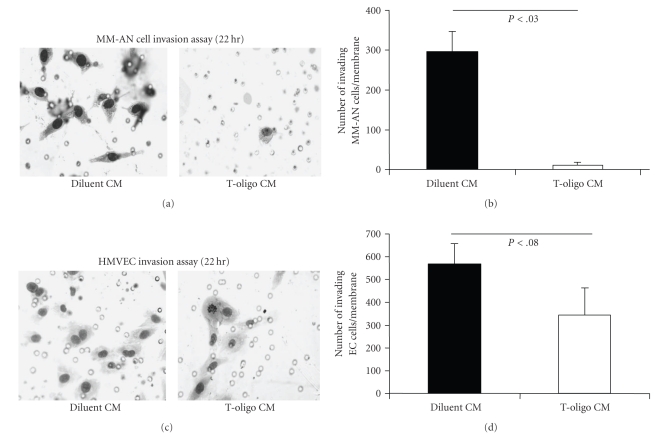
T-oligo treatment inhibits Matrigel invasion by MM-AN cells and HMVECs. MM-AN cells were treated with 40 *μ*M T-oligo or diluent alone. The culture medium was collected after 72 hours. The conditioned medium harvested after 72 hours was used as the chemoattractant for the invasion assay for MM-AN and HMVECs. MM-AN cells were plated on the inserts and allowed 22 hours to move through the pores on the membrane in the bottom of the inserts toward the medium in the lower chamber, interpreted as invasion of the gel. The experimental inserts had a layer of Matrigel, whereas control inserts (not shown) did not. After 22 hours cells that moved through the pores in the membrane were fixed, stained and photographed. (a) Representative images for MM-AN cells are shown. Small open circles are the pores in the membrane, not cells. (b) The total number of cells was counted for 3 membranes for each treatment condition and graphed as a number of cells (mean ± SEM) for both treatment groups. The assay was repeated twice with identical results. (c) Representative images for HMVECs treated as described for MM-AN above are shown. Small open circles are the pores in the membrane, not cells. (d) The total number of HMVECs were counted for 3 membranes for each treatment condition and graphed as an average number of cells for each treatment group (mean ± SD). The assay was repeated twice with identical results. Reductions approached but did not reach statistical significance.

We used MM-AN medium collected 72 hours after addition of T-oligo or diluent alone (MM-AN conditioned media: T-oligo CM or diluent CM) as the chemoattractant for an in vitro invasion assay [43]. MM-AN and HMVECs were both evaluated for invasion through Matrigel to determine if T-oligo treatment reduces the chemoattractant properties of MM-AN cells. As recommended by the manufacturer of the Matrigel invasion kit, after 22 hours of incubation the invading cells were fixed, stained and counted for each membrane. MM-AN cells plated above T-oligo CM had a ~96% decrease in invasion (*P* < .03) through Matrigel (Figures 3(a) and 3(b)). HMVECs plated on inserts coated with Matrigel and exposed to T-oligo CM had a ~40% reduction in Matrigel invasion compared to controls, but this did not reach statistical significance (*P* < .08) (Figures 3(c) and 3(d)). These findings demonstrate that T-oligos reduce the migration/invasion of MM-AN cells towards chemoattractant stimuli and, possibly, to a lesser degree affect HMVECs. This is consistent with the observation that MM-AN cells elaborate factors that promote migration and invasion of tumor cells, like VEGF and ANG-1, and that these factor(s) are reduced as a result of T-oligo treatment.

### 3.4. T-oligo Treatment Decreases the Expression of VEGF and VEGFR-2 in Endothelial Cells

**Figure 4:**
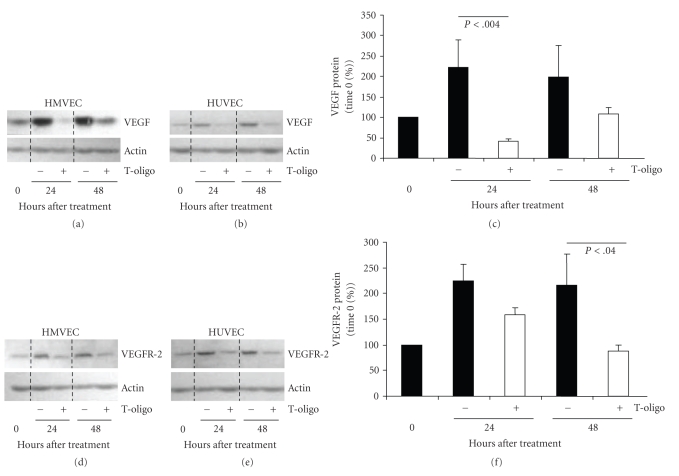
T-oligo decreases VEGF and VEGFR-2 protein levels in normal endothelial cells. HMVEC and HUVEC were treated with 40 *μ*M T-oligo or diluent alone and harvested for western blot analysis over 48 hours. (a) VEGF protein expression in HMVEC. Here and elsewhere actin expression was used to adjust the loading. (b) VEGF protein expression in HUVEC. (c) Combined densitometric analysis of VEGF expression in HMVEC and HUVEC as a percent of time 0 levels, after loading adjustment. (d) VEGFR-2 protein expression in HMVEC. (e) VEGFR-2 protein expression and HUVEC, and (f) Combined densitometric analysis, as in (c).

Because VEGF signaling through VEGFR-2 is principally responsible for EC survival, proliferation, migration, and angiogenesis [44–47], we examined the effect of T-oligo treatment on these proteins. HMVECs and HUVECs were grown and treated with 40 *μ*M T-oligo or diluent alone. Compared to diluent-treated control cells there was a decrease (*P* < .004) of ~86% in VEGF expression at 24 hours and an insignificant decrease of ~50% at 48 hours in T-oligo-treated samples (Figures 4(a)–4(c)). In addition, in T-oligo-treated HMVECs and HUVECs there were ~29% (*P* = NS) and ~59% (*P* < .04) reduction in the protein level of VEGFR-2 at 24 and 48 hours, respectively (Figures 4(d)–4(f)). Thus, T-oligo-mediated antiangiogenic effects on ECs may be, in part, due to inhibition of VEGF-VEGR2 axis.

### 3.5. T-oligo Inhibits EC Function In Vitro in Tubulogenesis Assay

**Figure 5:**
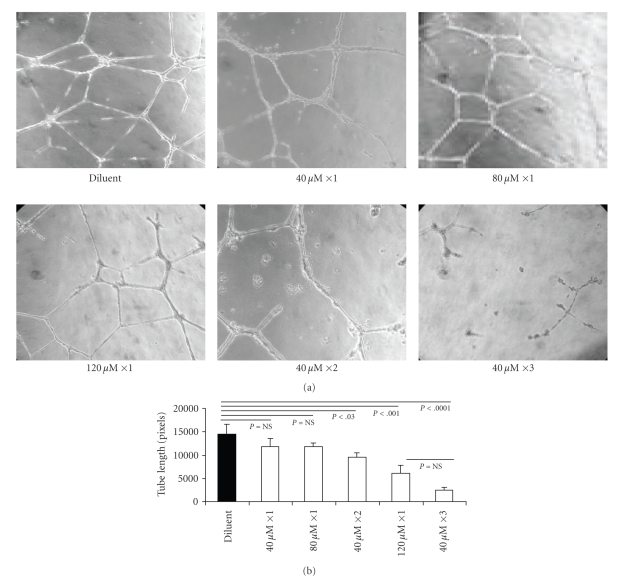
T-oligo treatment inhibits EC tubulogenesis in vitro. HMVEC cells were plated on Matrigel in four-well chamber slides and treated in triplicate with T-oligo or diluent alone, as described in the text. (a) All representative images are taken 22 hours after plating cells into chambers, the time of biggest differences among treatment conditions. (b) The length of tube-like structures was quantified as total average tube lengths per visual field from 3 separate chambers for each treatment condition. The differences in the length of tube-like structures were quantified (in pixels) in at least 3–5 representative photographs per chamber/treatment condition using computer-assisted image analysis.

HMVECs were plated on Matrigel in 4-well chamber slides in serum-containing medium and treated with diluent alone or increasing concentrations of T-oligo. Tube-like structure formation was maximal in controls at 22 hours and formal comparisons were made after 2–22 hours, as customary for this assay [39]. By 22 hours, in cells treated once with either 40 *μ*M or 80 *μ*M T-oligo there was a ~19% reduction in average tube length compared to cells treated with diluent alone (*P* = NS) (Figures 5(a) and 5(b)). Cells treated with two separate doses of 40 *μ*M T-oligo at plating and 2 hours after plating showed a ~35% reduction in average tube length (*P* < .03). Cells treated once at plating with 120 *μ*M T-oligo showed a ~58% reduction in average tube length compared to the diluent-treated cells (*P* < .001). A reduction of ~83% (*P* < .0001) in tube length was observed in cells treated with 3 separate doses of 40 *μ*M T-oligo (40 *μ*M ×3) added to the medium at the time of plating and then at 2 and 6 hours (Figures 5(a) and 5(b)). These results indicate that T-oligo inhibits EC function in a manner dependent on the dose and frequency of administration, with the caveat that the very large amount of oligos administered, rather that specific telomere-based T-oligo initiated signalling may have contributed to the effect observed. In our future experiments, we plan to examine the effect of large and/or fractioned T-oligo doses on survival of ECs.

### 3.6. Systemic Administration of T-oligo Reduces Tumor Angiogenesis and Vessel Patency In Vivo in a SCID Mouse Xenograft Model

**Figure 6:**
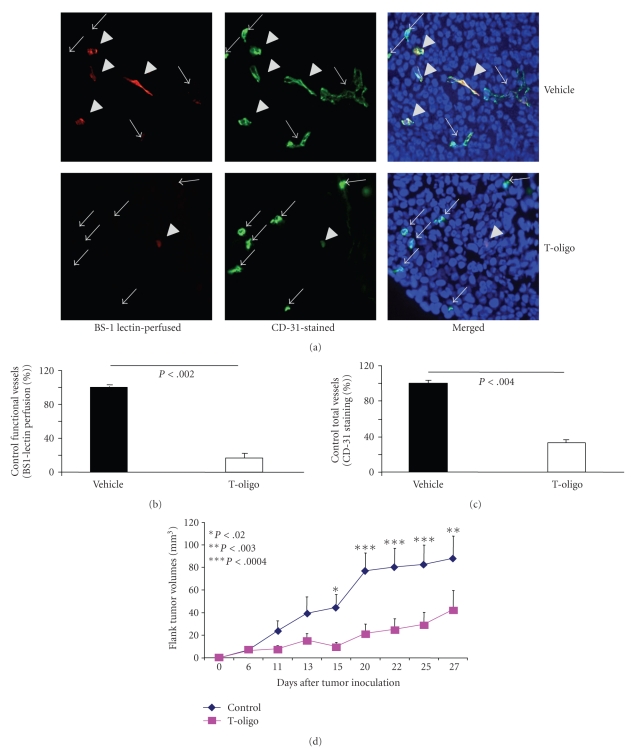
T-oligo decrease tumor angiogenesis and melanoma tumor volumes in mouse SCID xenografts. (a) Representative images of 6 *μ*m tumor cross-sections immunostained with CD31 (green) and TopRo-3 (blue-nuclei) and perfused in vivo with BS-1 lectin (red), to determine tumor microvascular density (MVD) per high power field (HPF) ×40 magnification. Both functional and total vessels were examined in 5 mice/group. Arrows indicate CD31 (+) vessels that are considered nonfunctional (not perfused) whereas arrowheads indicate double (+) BS-1 lectin/CD31 vessels that are considered functional (perfused in vivo). (b) Percent functional vessels (red-BS-1 lectin staining) in T-oligo injected mice, taking MVD in vehicle injected mice as 100%. (c) Percent total vessels (green-CD31 staining) in T-oligo injected mice, taking MVD in vehicle injected mice as 100%. (d) SCID mice were injected with MMAN cells into the flank. T-oligo or vehicle was injected daily for up to 5 days when tumors were first palpable (2-3 mm diameter). Average tumor volume/animal was recorded over 4-weeks in 5-6 mice/group.

In melanoma tumors inoculated into SCID mice, we determined the effect of systemic T-oligo injection on melanoma angiogenesis by evaluating functional and total vessel density in tumor tissue (5 mice per group). When tumors became palpable (2-3 mm in diameter, day 5 to 14 after inoculation) we injected T-oligo (60 nmoles/injection, 15 mg/kg) via the tail vein (IV) and injected again after 6 hours, then harvested tumors 24 hours after the second T-oligo injections. Representative images of tumor cross-sections were immunostained to identify all vessels as well as functional (patent) vessels 24 hours after T-oligo injection (Figure 6(a)). Compared to control-injected mice there was more than ~80% decrease (*P* < .002) in functional vessels in T-oligo-injected mice (Figure 6(b)). There was also more than ~60% decrease (*P* < .004) in total vessels in T-oligo-injected mice (Figure 6(c)). These data corroborate our in vitro findings (Figures 4 and 5) and demonstrate that two systemic administrations of T-oligo reduce tumor angiogenesis and vessel patency in vivo (Figures 6(a)–6(c)). 

**Figure 7:**
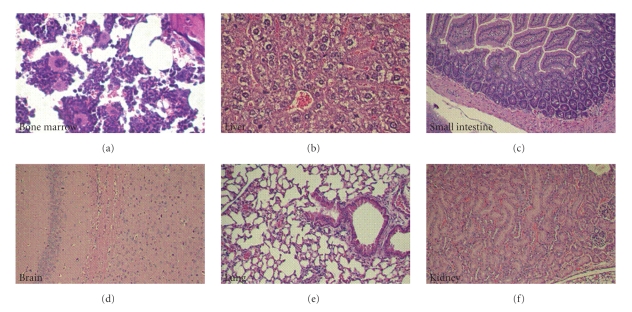
Evaluation of T-oligos toxicity in internal organs of SCID mice 24 hours after the last IV injection (15 mg/kg BID for 5 days). (a) Bone marrow, displaying a mixture of myeloid and erythroid precursor cells as well as plasma cells. Scattered megakeryocytes are also present. There is no evidence of bone marrow suppression or toxicity. (b) Liver lobule with a central vein surrounded by hepatocytes. The cells display a fixation artifact but otherwise appear normal. There is no evidence of cellular necrosis or apoptosis. (c) Jejunal mucosa displaying normal arrangement of villi lined by tall columnar cells. Both the mucosa and the submucosa appear normal. Fragments of normal pancreatic acinar tissue are seen in the bottom left of the image. (d) Section of the brain showing normal brain architecture with typical neuronal ganglia and scattered small dark glial cells in a pink neuropil background. (e) Normal lung tissue displaying multiple alveoli as well as bronchioles lined with cuboidal epithelial lining. (f) The kidney displays two normal glomeruli that are surrounded by tubules with cuboidal epithelium.

To determine the effect of systemic T-oligo treatment on melanoma growth, in SCID xenograft model MM-AN cells were injected subcutaneously in the flank. When tumors became palpable (2-3 mm in diameter, day 5 to 14), the mice received daily systemic injections of T-oligo (60 nmoles/injection, 15 mg/kg BID) or vehicle for 5 days only. In control animals, tumors increased in volume from 6.96 ± 2.9 mm^3^ on day 6 to 88.04 ± 19.72 mm^3^ on day 27 (Figure 6(d), blue line). In contrast, in T-oligo treated animals, whose tumors were the same size as in controls on day 6 (6.48 ± 2.57) there was very little tumor growth through day 15 (6.48 ± 2.57 versus 9.92 ± 3.8 mm^3^) and thereafter a slower and statistically insignificant increase in volume to 29.65 ± 10.47 mm^3^ by day 27 **(** Figure 6(d), pink line**)**. Thereby, in T-oligo-treated mice tumor growth was inhibited (*P* < .003) by ~53% after ~4 weeks, when the experiment was terminated. These data demonstrate that systemic administration of T-oligo for only 5 days has a persistent inhibitory effect on melanoma growth. We have also evaluated T-oligos toxicity in several internal organs of SCID mice 24 hours after the last IV injections (15 mg/kg BID for 5 days). No systemic toxicity was observed in bone marrow, liver, intestines, brain, lungs and kidneys of T-oligo-injected mice (Figures 7(a)–7(f)).

## 4. Discussion

Tumor angiogenesis is essential for tumor growth and metastasis [1]. Without active angiogenesis tumor diameter rarely exceeds 2-3 mm [1, 48]. Angiogenesis is mediated through release of angiogenic factors by tumor cells and cells in the tumor stroma and microenvironment including, but not limited to, endothelial cells [48]. We now report that telomere homolog oligonucleotides (T-oligos) decrease the synthesis and release of angiogenic factors by ECs and melanoma cells, inhibit EC tubulogenesis and impede melanoma cells and ECs from invading matrix (Matrigel). In addition, systemic administration of T-oligos decreases tumor vascularity in vivo.

EC migration, proliferation and differentiation are all essential processes for tumor angiogenesis [1]. EC proliferation, in vitro tubulogenesis, and survival are all known to be stimulated in large part by VEGF [49]. Decreased VEGF levels or inhibition of receptor activation in ECs often correlate with decrease in tumor size and metastatic potential [50]. VEGF binds to the extracellular domain of the VEGFR-1 (Flt-1) and VEGFR-2 (Flk-1), inducing receptor dimerization and activation of tyrosine kinases by autophosphorylation, leading to angiogenesis, increased vascular permeability, and EC proliferation and survival [49]. It is generally accepted that VEGFR-2 is the major mediator of these effects [51]. We found that T-oligo decreases the expression of VEGFR-2 by HMVEC and HUVEC (Figures 4(c) and 4(f)). Other investigators reported that receptor tyrosine kinase inhibitors (TKIs) such as sunitinib and dasatinib reduce signaling through the RAF/MEK/ERK pathway that is activated by ligand binding to angiogenic receptors like VEGFR-2, PDGFR-*β*, FH-3 and c-kit [52], indirectly inhibiting tumor growth by affecting tumor angiogenesis [52]. By reducing VEGF-R2 level, T-oligos appear to have similar effects.

VEGF also induces leakage within tumor vessels, allowing tumor cells to infiltrate blood vessels and migrate into the blood stream [53]. Hence, changes in angiogenic factors even early in tumor formation can affect metastasis and spread [53] and inhibiting VEGF production by T-oligos would be expected to reduce the metastatic potential of tumor cells [53]. Additionally, increased blood vessel permeability within the tumor may interfere with adequate delivery and retention of chemotherapeutic agents [54, 55]. Indeed, certain antiangiogenic agents that prevent tumor vessel leakage (a phenomenon called “vessel normalization”) were shown to enhance the delivery of chemotherapeutic agents into tumors [56]. Thus, combination treatment with antiangiogenic factors together with conventional chemo-therapeutic agents may be superior to using the latter alone. Furthermore, because VEGF is likely required for migration and recruitment of ECs, T-oligo-me diated VEGF reduction would also likely decrease the number of blood vessels in the tumor [1, 6]. Our present findings suggest that T-oligos may induce potent antiangiogenic effects, enhancing their attractiveness as a therapeutic option for cancer.

In addition to VEGF and its receptors, angiopoietin 1 and 2 (ANG-1 and ANG-2) and their tyrosine kinase receptor Tie-2 have been identified as major players in the processes of growth and remodelling of tumor vasculature [57, 58]. ANG-1 activates the Tie-2 signalling pathway [58]. Although under certain conditions ANG-2 may inhibit ANG-1 effect, in an in vivo mouse model for melanoma [59] and in glioma cells [60] increased expression of ANG-2 is thought to stimulate angiogenesis. Another angiogenic factor, HIF-1*α*, a transcription factor that is activated by hypoxia, exerts its effect by upregulating VEGF levels. We found that T-oligo treatment inhibits HIF-1*α* activity and ANG-1 and ANG-2 expression in melanoma cell line and decreases VEGF synthesis secretion in these cells, strongly suggesting that T-oligo-mediated effects on tumor angiogenesis are transcriptional and ultimately affect several angiogenic molecules.

Our laboratory has previously shown that T-oligo increases p73 level in the p53 null MM-AN cells and that blocking p73 expression by RNAi decreases T-oligo-induced apoptosis in these cells [11]. Like p53, p73 is known to inhibit angiogenesis, primarily through VEGF down-regulation [34]. Therefore, we assume that in MM-AN cells T-oligo decreases VEGF production, in addition to its effect on HIF-1*α*, by activating p73 through induction of E2F1. However, in cells with functional p53, we assume that T-oligo-induced p53 and p73 would cooperate to inhibit angiogenesis. Furthermore, E2F1 is known to induce apoptosis in part by its effect on p73. Thus, induction of E2F1 by T-oligos [11] also contributes to tumor cell apoptosis. E2F1 is reported to be upregulated by active (phosphorylated) ATM [61]. We have previously shown that T-oligo treatment activates (phosphorylates) ATM [21, 27]. Moreover, the E2F1 promoter contains binding sites for E2F1 [62]. We therefore suggest that T-oligo regulates E2F1 first via ATM-mediated phosphorylation of E2F1 [61] and that E2F1 then further transcriptionally upregulates E2F1 by binding to its own promoter. Indeed, we show that T-oligo increases E2F1 mRNA and protein levels as well as its DNA binding activity in human MM-AN melanoma cells, further confirming our previous finding that E2F1contributes to T-oligo effects [11]. 

The T-oligos used in the present experiments have physiologic readily hydrolysable phosphodiester linkage, unlike for example antisense DNA in which phosphorothioate or other nonhydrolyzable linkage is employed to increase the molecule's half-life (T 1/2). Hydrolyzable linkage is required for initiation of T-oligo signalling [22] and, despite the known short T 1/2, approximately 4–6 hours in serum-containing medium for a 12-base 100% telomere homolog [38], nevertheless this allows for the cellular responses observed in the present and previous experiments to evolve over 3–5 days [11, 13, 19, 21, 22, 24–28]. This may reflect the fact that at least in vitro T-oligos rapidly enter the nucleus [11, 22, 24, 27] and that, once in the nucleus, such oligos have a far longer T 1/2 [63]. The efficacy of these presumptively short-lived T-oligos may also reflect the likelihood that, after interaction of the oligos with the Werner protein and formation of DNA damage-like foci at telomeres [22], signalling through the DNA damage response pathways may continue even if the T-oligos have been hydrolyzed, at least through 48 hours at which time the DNA damage foci can still be observed by immunohistochemistry [22].

In earlier studies, we have shown that T-oligos inhibit tumor growth in SCID mouse models by inducing cell cycle arrest, differentiation, apoptosis, and senescence [13, 15, 27]. We now show that T-oligos also inhibit angiogenesis. Angiogenesis inhibition encompassing both ECs and melanoma cells, in combination with other T-oligo-mediated anti-tumor effects [11, 13, 22, 25, 27], likely combine to significantly decrease melanoma burden in established SCID mouse models, as reported in our earlier publications [13, 27]. These multiple diverse responses are all mediated through activation and/or upregulation of DNA damage response proteins. They are thus reminiscent of the less complex but very well characterized bacterial SOS response, a genome-protective mechanism that enhances survival of prokaryotic organisms in the face of DNA damage [14]. The telomere-based DNA damage-like signalling initiated by T-oligos may be viewed as an evolutionarily perfected parallel mechanism in mammalian cells that addresses the threat to genomic integrity posed by malignant transformation [64].

In summary, the present paper indicates that T-oligos exert multiple antiangiogenic effects. These data reinforce prior evidence that T-oligo therapy may offer a powerful new approach to treatment of human primary melanoma and possibly other human malignancies, with several conceptual advantages over the currently lionized targeted therapy approach [64].
